# Empowering Leadership and Unethical Pro‐Organizational Behavior: A Social Exchange Perspective in Uncertain Environments

**DOI:** 10.1002/pchj.70064

**Published:** 2025-11-04

**Authors:** Yang Jia, Chao Pan

**Affiliations:** ^1^ The Tourism College of Changchun University Changchun China; ^2^ School of Public Affairs, Zhejiang University Hangzhou China

**Keywords:** empowering leadership, environmental uncertainty, leader‐member exchange, social exchange theory, unethical pro‐organizational behavior

## Abstract

Previous literature has overlooked the impact of environmental factors on the effectiveness of empowering leadership, and this study introduced the important boundary factor of environmental uncertainty as a moderating role, and constructed a moderated mediation model to investigate how empowering leadership influences employees' unethical pro‐organizational behavior from a social exchange perspective. We collected data at three different time points and administered questionnaires to 431 employees. Hypotheses were tested in PROCESS using the bootstrapping method. The results showed that: (1) There was a positive relationship between empowering leadership and unethical pro‐organizational behavior, and leader‐member exchange played a mediating role in this relationship. (2) Environmental uncertainty played a moderating role between empowering leadership and leader‐member exchange. With the increase of environmental uncertainty, the positive relationship between the two is weakened. This study contributes to leadership literature by integrating environmental uncertainty into the social exchange framework, highlighting its impact on leader‐member exchange and unethical pro‐organizational behavior, thereby offering fresh insights into leadership effectiveness in dynamic environments.

## 1 Introduction

As the economist Friedman explained, the instability and unpredictability of globalization and technological change make even the most capable leaders obsolete in this dynamic environment (Freidman 2005). For a long period of time in the past, the external environment of organizations was relatively stable, and the internal mobility of organization members was also low, so less research was focused on and discussed on the effectiveness of leadership. Since the beginning of the 21st century, with the help of information technology, global competition has intensified, and the factors affecting organizational development have become increasingly complex and uncontrollable. The uncertain environment has posed unprecedented challenges to the effectiveness of leadership (Cicero et al. 2010; Rast III 2015). Therefore, some researchers believe that it is necessary to include environmental factors in the study of the effectiveness of leadership models (Cheong et al. 2019).

As the competitive environment in business becomes increasingly intense, many organizations are forced to transform their internal management models to adapt to this complex and highly uncertain environment. For example, to maximize efficiency, organizations have been flattening their organizational structures by expanding employees' responsibilities and scope of work (Biemann et al. 2015). In this process, empowering leadership (EL) plays a key role because of its characteristics in promoting employee self‐management and reducing unnecessary organizational norms for the employee (Lee et al. 2018). Despite the obvious benefits that EL brings to organizations in an uncertain business environment, it may not be the same for employees. An uncertain environment represents a lack of control and unknown risks, and employees may struggle to bear the risks of making mistakes when authorized to make some decision‐making work in such an environment (Twyford and Le Fevre 2019). Therefore, environmental uncertainty (EU) may be an important challenge to the effectiveness of EL, and the aim of this study is to verify this possibility.

This study takes a social exchange perspective to explore the impact of EL on employees' work outcomes. According to social exchange theory, there is a reciprocity principle in social interactions; that is, individuals adjust their responses to others according to the material or non‐material levels of others' contributions to individuals (Cropanzano and Mitchell 2005). However, research on social exchange in the field of organizational management has ignored environmental factors. This study responded to the call made by Cheong et al. (2016) to consider boundary conditions that may alter the relationship between authorized leadership and work‐related outcomes, thus integrating EU, an environmental factor, into the framework of social exchange theory and combining it with the EL literature, thereby opening up a new agenda for future research on the effectiveness of EL.

### 1.1 Empowering Leadership and Unethical Pro‐Organizational Behavior

EL is defined as allocating autonomy and responsibilities to individual employees or teams from the leaders, resulting in employees' promoted internal motivation and external performance (Cheong et al. 2019). As defined, EL is bound to have a series of psychological and behavioral effects on employees, and numerous studies have confirmed this view. For instance, EL could have a positive impact on employees' job satisfaction (Amundsen and Martinsen 2015; Kim et al. 2018), job performance (Ahmed et al. 2022; Wang et al. 2022), and innovative behavior (Guo et al. 2023; Rao Jada et al. 2019). However, recent research has also found that the effectiveness of EL is not universally supported (Cheong et al. 2019; Hassan et al. 2013). Some researchers believe that EL may lead to potential negative effects, which will have adverse effects on employees and organizations (Cheng et al. 2024; Lee et al. 2017). For example, Cheong et al. (2016) found that EL objectively increases the workload of subordinates, which leads to work stress and a decrease in their work performance. Other researchers also found that EL would intensify employees' perception of leadership expectations, which would require them to work hard to prove their worth, leading to controlled work emotions and further reducing employee performance (Hao et al. 2018). Therefore, EL is a double‐edged sword, but the discussion of its negative impacts is still insufficient compared to its positive effects.

Unethical pro‐organizational behavior (UPOB) refers to actions that are intended to benefit the organization potentially while violating core social values (Umphress and Bingham 2011), which is an interesting topic, because it represents a dilemma faced by employees, where they must choose between benefiting the organization and adhering to universal ethical standards. There are many explanations for UPOB, and two of the most prominent perspectives are the social identity theory and the social exchange theory (Mishra et al. 2021). The former view is that employees who have a high level of organizational identification will prioritize organizational interests over the interests of victims of unethical behavior because a strong sense of organizational identification will prompt them to ignore ethics and view UPOB as responsible behavior (Effelsberg et al. 2014; Umphress and Bingham 2011). The latter view is based on the principle of reciprocity in interpersonal relationships, which suggests that employees try to use UPOB as a means of repaying the favor and benefits they have received from their superiors and the organization in order to establish long‐term social exchange relationships characterized by mutual trust and respect (Wang et al. 2019). Therefore, some scholars believe that individuals who have positive social exchange relationships with their employers are more likely to engage in UPOB (Umphress and Bingham 2011).

Currently, UPOB has been proven to be closely related to leadership (Manelkar and Mishra 2024; Mishra et al. 2021). As for the relationship between EL and UPOB, we believe that the social exchange perspective is more appropriate among the two prominent explanatory mechanisms of UPOB. Because the core of social identity theory focuses on the belonging relationship between individuals and groups as well as the consensus at the group level, while the core of social exchange theory is that individuals maintain relationships through “giving and receiving” exchanges, focusing on the dynamic balance in specific interactive relationships, which is highly consistent with the core attributes of leader‐member exchange (LMX). In other words, the social exchange theory can precisely explain the mediation mechanism of LMX, while the social identity theory cannot. According to the social exchange perspective, the power and opportunity granted to employees by EL to participate in decision‐making are viewed by employees as a motivational factor, representing respect and trust from the superior (O'Donoghue and Van Der Werff 2022). When leaders express confidence in their employees' abilities and grant them partial autonomy, employees feel obliged to choose the best attitudes and behaviors to demonstrate their abilities in order to reciprocate the trust that the leaders have shown in their abilities (Rai and Kim 2021). However, this “repay” for EL may not necessarily lead to good behavior outcomes. Because individuals with higher task autonomy will have more opportunities to participate in task‐related decision‐making, thus requiring additional, differently categorized information processing, which will increase their cognitive interference and role stress (Cheong et al. 2019; Langfred and Moye 2004), causing incorrect decisions to be made during the work process, such as ignoring unethical attributes and only considering pro‐organizational attributes of UPOB. Therefore, we suggest that:

Hypothesis 1 *There is a positive relationship between EL and UPOB*.

### 1.2 The Mediating Role of Leader‐Member Exchange

LMX refers to the mutual relationship that is established between the leaders and employees during the work process and emphasizes the quality of relationships between them (Liden et al. 1993; Martin et al. 2018). The establishment of this relationship requires the leader to provide subordinates with certain benefits, such as fair treatment, support, or autonomy (Liang et al. 2022; Matta et al. 2024; Volmer et al. 2012). Under this premise, EL seems to be the best choice for improving leader‐employee relations, as evidenced by previous research (Amundsen and Martinsen 2014; Lee et al. 2018). Specifically, from a social exchange perspective, when leaders emphasize the importance of the employees' work and allow them to participate in decision‐making, it expresses trust in the employees, which can be seen as a benefit that leaders give to employees (Cheong et al. 2016). It may enhance employees' own perceptions of their impact on the organization and encourage them to pay attention to their roles in the organization. Then, LMX is promoted when employees attempt to reciprocate leaders' trust and establish further positive exchanges with leaders and organizations (Kwak and Jackson 2015).

Although previous studies have had some discussions about the relationship between EL and LMX, LMX and UPOB respectively (Amundsen and Martinsen 2014; Li et al. 2024), this study will for the first time validate the mediating role of LMX between EL and UPOB from a social exchange perspective. Specifically, employees may recognize EL as the signal that the leader is committed to a long‐term relationship, which in turn creates more socio‐emotional connections, like LMX (Cheong et al. 2016). Employees who perceive high‐quality LMX tend to have higher levels of belonging, responsibility, and organizational commitment (Chen et al. 2008; Eisenberger et al. 2010), and are therefore more willing to put in extra effort and demonstrate higher levels of job performance and extra‐role behavior (Martin et al. 2016). Meanwhile, they will feel that they are more likely to receive trust and support from their leaders or the organization, may enjoy additional privileges and protection, and thus have a stronger sense of psychological security (Hu et al. 2018; Venkatesh et al. 2023).

In return for the leaders, they are also willing to take risks and do their best to assist the leadership in their work, even going beyond their assigned tasks. However, UPOB is very likely to occur during the LMX process. The possible reasons might be: First, it is very easy to trigger the moral licensing effect in this process (employees may feel entitled to engage in unethical behavior, such as UPOB, after performing ethical behaviors such as working overtime or making sacrifices for the team) (Mishra et al. 2021). Second, emotional connection leads to the blurring of moral boundaries. Employees will extend their emotional obligations to the leader into special responsibilities to the organization and tacitly assume that actions that do not disappoint the leader are in line with the organization's interests, thereby weakening their independent judgment on the morality of behavior (Bryant and Merritt 2021). Finally, trust spillover leads to the expectation of risk sharing (Chen et al. 2015), that is, high‐quality LMX makes employees have the risk perception that “the leader will take the safety net for me.” Conversely, in low‐quality LMX, employees have difficulty perceiving their leaders' recognition and support. As a result, they are unwilling to take risks for their leaders or do extra work to repay their leaders (Matta et al. 2024), so they may have a lower UPOB. Therefore, we propose that:

Hypothesis 2 *LMX plays a mediating role between EL and UPOB. Specifically, EL promotes an emotional connection with its employees through trust and empowerment, namely LMX. This high‐quality connection may drive employees to implement UPOB through mechanisms such as moral permission, emotional obligation, and trust spillover*.

### 1.3 The Moderating Role of Environmental Uncertainty

EU refers to the degree to which individuals perceive it difficult to accurately predict the future state of the external environment (López‐Gamero et al. 2011). As an essential characteristic of the organizational environment, EU has significant effects on the psychological behavior and motives of leaders and employees (Shirokova et al. 2016). Meanwhile, EU has posed new chances and challenges to the effectiveness of EL. From a leadership and organizational perspective, the benefits of EL in dynamic environments can be more pronounced, as in fast‐changing and uncertain business environments, organizations need to be flexible in responding to market changes, and EL allows employees to have more autonomy, enabling them to quickly adjust their work methods, innovate and solve problems, thereby helping organizations better respond to external challenges (Carmeli et al. 2011; Hmieleski and Ensley 2007; Sharma and Kirkman 2015).

However, from the employee's perspective, the situation reverses. Because in uncertain environments, individuals perceive higher levels of risk, and to avoid this risk, they reduce their proactive risk‐taking behavior and seek more additional information and feedback (Foster‐Fishman and Keys 1997; Viscusi et al. 1991). As for employees, they will feel more insecure when they perceive an uncertain environment (Glambek et al. 2023; Lian et al. 2022), and this insecurity will increase their stress and anxiety (Cheng and McCarthy 2018). In this situation, they may prefer clear guidance rather than more autonomy granted by EL. For example, after conducting a survey of the reform measures of a large service organization, Foster‐Fishman and Keys (1997) found that measures that conflicted with the organization's culture brought about ambiguity and dynamism. Despite the good intentions of the organizational leadership and the opportunities for employees to participate in decision‐making, most employees rejected these new opportunities and did not trust the intentions of the leaders. Therefore, this study suggests that when the environment is highly uncertain, EL may actually exacerbate employees' anxiety in such situations because employees lack the necessary resources and information to make effective decisions, leading to delegation behaviors that fail to meet their actual needs. In other words, although EL has a natural advantage in increasing LMX, in high EU situations, the empowering behavior of EL may backfire, even weakening employees' trust in the leader and affecting the quality of LMX. Based on this, we propose that:

Hypothesis 3 *EU plays a negative moderating role between EL and LMX. With the increase of EU, the positive relationship between EL and LMX is weakened*.

**FIGURE 1:**
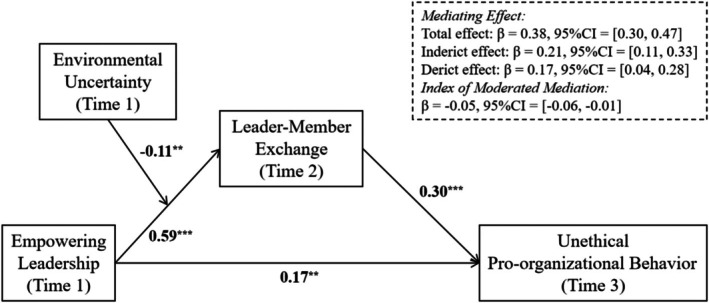
Hypothesis model in this study.

Overall, the model in this study showed in Figure 1.

## 2 Method

### 2.1 Participants and Procedure

A questionnaire survey was adopted in this study, and the samples were collected from dozens of enterprises in Henan Province, China. Henan Province is the province with the largest population in China, which is why labor‐intensive industries such as manufacturing are very developed, including new energy vehicles and smartphones. In addition, researchers have previously investigated this site (Li et al. 2018). In this context, the participants in this study were all from the manufacturing industry of Henan Province. The data collection was completed with the consent of the person in charge of the enterprise and the human resources department. To minimize common method bias, the study was completed in three time points with a one‐month interval between each time point (Podsakoff et al. 2012). At time 1 (MAR, 2024), we directly collected offline data on EL and EU from 715 employees (the valid data was 644). At time 2 (APR, 2024), the data on LMX was collected (the valid data was 502). At time 3 (MAY, 2024), the data on UPOB and demographic information were collected (the valid data was 431). According to the recommendations of previous researchers (DeSimone et al. 2015), the criteria we used to screen effective questionnaires were: incomplete response (i.e., some options have not been filled in), lengthy strings of invariant responses (i.e., the same option being selected repeatedly), and more importantly, failed the attention test (i.e., an additional item was set up, called “Please select ‘Most Disagree’ option for this question.” And if this option is not selected, we assume that this participant was not paying attention while taking this survey). Finally, 431 valid data were collected (60.2%). There were 282 males (65.4%) and 149 females (34.6%). The mean age was 37.78 years (SD = 9.77). All participants agreed to participate in the study and received a ¥10 payment at the end.

### 2.2 Measures

To ensure the quality of the scales, only those scales were chosen that had been validated in important international journals, and all scales were subjected to a two‐way translation process to ensure accuracy by bilingual experts (Ozolins et al. 2020). More importantly, these scales have corresponding Chinese versions, which have been well validated in other research (Cheng 2017; Li et al. 2017; Su et al. 2010; Zhang 2020).

*Empowering Leadership Scale*: We used the EL scale with 12 items (Ahearne et al. 2005), which is rated on a five‐point Likert scale. An example item is “My manager makes many decisions together with me.” In this study, the Cronbach's α was 0.94.

*Environmental Uncertainty Scale*: We used the EU scale with 3 items (De Hoogh et al. 2005), which is rated on a seven‐point Likert scale. An example item is “To which degree is your work environment dynamic.” In this study, the Cronbach's α was 0.93.

*Leader‐Member Exchange Scale*: We used the LMX scale with 7 items (Scandura and Graen 1984), which is rated on a four‐point Likert scale. An example item is “How certain are you that your leader is satisfied with your performance.” In this study, the Cronbach's α was 0.93.

*Unethical Pro‐organizational Behavior Scale*: We used the UPOB scale with 6 items (Scandura and Graen 1984), which is rated on a five‐point Likert scale. An example item is “If it helps the company, I will distort the facts to maintain the image of the organization.” In this study, the Cronbach's α was 0.82.

*Control Variables*: Based on the previous studies (Dennerlein and Kirkman 2022), we asked the employees to provide the following demographic information: gender, age, education, position, and working duration with their superior as control variables, as shown in Appendix A (Table A1).

### 2.3 Data Analysis

We analyzed the data using SPSS 26.0 and Mplus 7.0. First, the confirmatory factor analysis was conducted by Mplus. Second, descriptive statistics and a correlation matrix were performed. Finally, the hypothesized moderated mediation model was tested using Model 7 of the Process 3.3 procedure in SPSS (Hayes et al. 2017).

## 3 Results

### 3.1 Common Method Bias Test

This study specially adopted multiple time points for data collection, and used common latent factor (CLF) analysis to test common method bias. According to the suggestion of Podsakoff et al. (2012), after we added a common latent factor, although *χ* ^*2*^ and df were slightly improved, the variation of fitting indicators such as CFI, TLI, SRMR, and RMSEA ranged only from 0.001 to 0.1 (see Table 1). Therefore, we believe that although the common method bias problem is smaller in this study and is still foreseen to have a minor impact on the research results, we believe that this impact is acceptable.

**TABLE 1:** Fit indices of different models.

Models	Factor	*χ* ^2^	df	CFI	TLI	SRMR	RMSEA
M1	EL, EU, LMX, UPOB, CLF	1301	343	0.91	0.91	0.05	0.06
M2	EL, EU, LMX, UPOB	1382	344	0.90	0.91	0.05	0.07
M3	EL + EU, LMX, UPOB	2421	347	0.78	0.76	0.08	0.12
M4	EL + EU + LMX, UPOB	3148	349	0.70	0.67	0.09	0.14
M5	EL + EU + LMX + UPOB	3815	350	0.63	0.60	0.11	0.15

*Note:* EL = Empowering leadership, EU = Environmental Uncertainty, LMX = Leader‐Member Exchange, UPOB = Unethical Pro‐organizational Behavior. M1 is a model with four factors that are mutually independent and have a common latent factor added; M2 is a model with four independent factors; M3 is a model in which EL and EU are combined, with other factors being independent; M4 is a model in which EL, EU, and LMX are combined, with UPOB being independent; M5 is a model in which all four factors are combined together.

### 3.2 Reliability and Validity Test

First, internal consistency reliability (Cronbach's α) was assessed for each scale. The results indicated that Cronbach's α for EL, EU, LMX, and UPOB were 0.94, 0.93, 0.93, and 0.82 respectively, exceeding 0.8 and demonstrating excellent reliability. Then, we used Mplus to conduct confirmatory factor analysis. We first sorted out the original survey data, including coding and standardization of all variables, and then we chose the maximum likelihood estimation (ML) method in Mplus. By comparing the fit indices of the different models, it was found that the fit indices of the M2 (*χ* ^*2*^ = 1382, df = 244, CFI = 0.90, TLI = 0.91, SRMR = 0.05, RMSEA = 0.07) were better than those of M3–5, indicating better discriminant validity of the variables. The findings are summarized in Table 1.

### 3.3 Correlation Analysis

**FIGURE 2:**
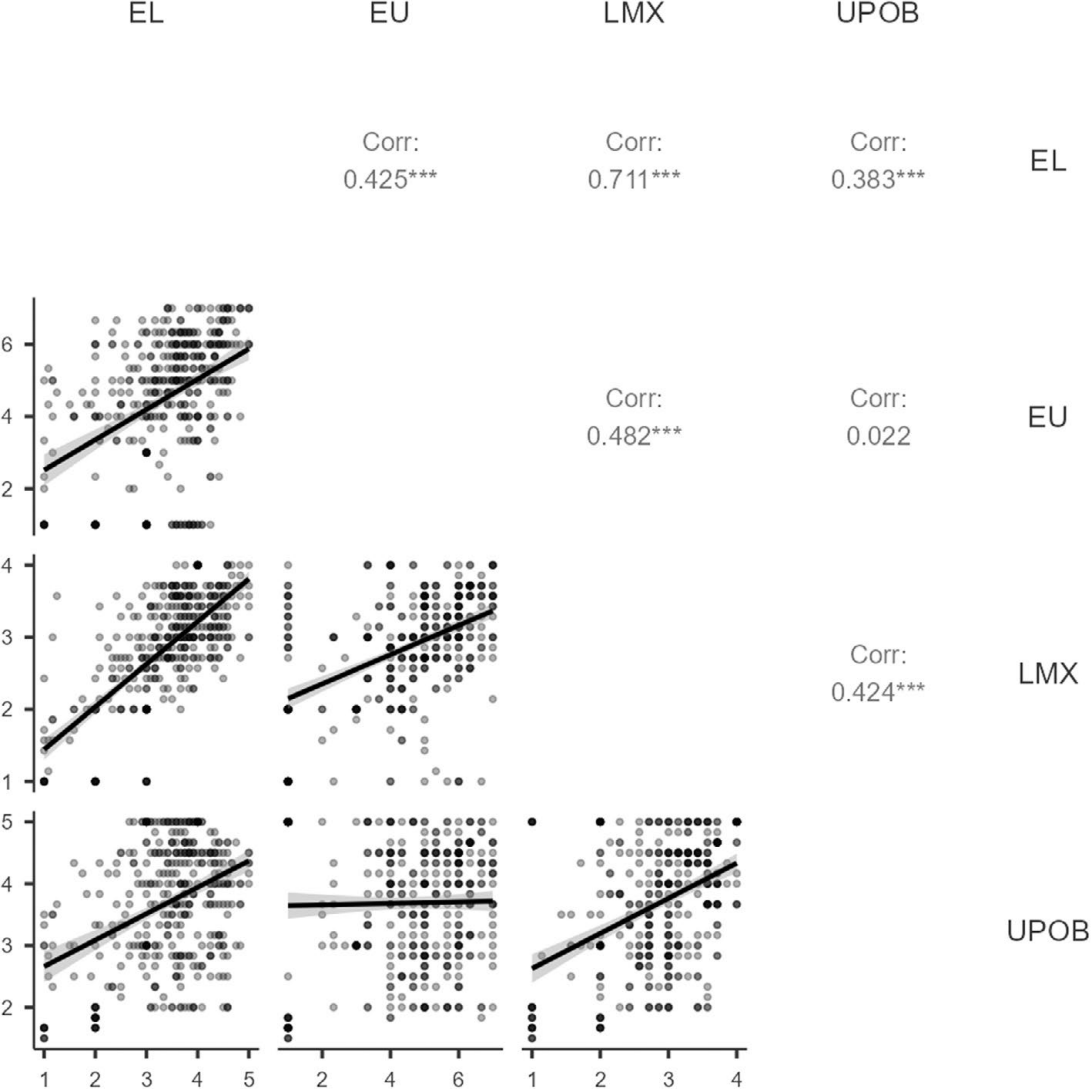
Correlation matrix and scatter plot. EL = Empowering leadership, EU = Environmental Uncertainty, LMX = Leader‐Member Exchange, UPOB = Unethical Pro‐organizational Behavior. ***p < 0.001.

The correlation analysis (see Figure 2) found that EL was significantly positively correlated with LMX and UPOB. Furthermore, LMX was significantly positively correlated with UPOB. The findings are summarized in Figure 2.

### 3.4 Hypothesis Test

We first built a moderated mediation model using Model 7 in the Process procedure of SPSS. After controlling the variables like gender, age, education, position, and working duration with their superior, the results showed that EL and UPOB had a significant positive relationship (β = 0.17, SE = 0.06, *t* = 2.66, *p* < 0.01), supporting H1. The interaction item of EL and EU had a significant relationship with LMX (β = −0.11, SE = 0.03, *t* = −3.50, *p* < 0.01), supporting H3. Additionally, based on bootstrap analyses with 5000 repetitions, it was found that LMX played a mediating role between EL and UPOB (β = 0.22, SE = 0.06, 95%CI = [0.11, 0.33], *p* < 0.001), verifying H2. It is worth noting that the control variables seem to have no significant impact on LMX and UPOB. The possible reasons are that the core variables have a stronger influence on the explained variables. In addition, the samples are all from the manufacturing industry and have relatively high homogeneity. The detailed results are presented in Figure 1 and Table 2.

**TABLE 2:** Results of regression analysis.

Variables	LMX			UPOB		
	β	SE	*t*	β	SE	*t*
Control Variables						
Gender	−0.12	0.07	−1.65	0.03	0.09	0.31
Age	0.00	0.00	−0.83	−0.01	0.00	−1.84
Education	0.02	0.04	0.49	−0.04	0.05	−0.87
Position	0.01	0.03	0.34	0.02	0.05	0.52
Working duration with their superior	0.00	0.00	0.80	0.00	0.00	1.82
Independent Variable						
EL	0.59	0.03	16.43***	0.17	0.06	2.66**
Moderating Variable						
EU	0.21	0.04	5.83***			
Interaction Item						
EL × EU	−0.11	0.03	−3.50**			
Mediating Variable						
LMX				0.30	0.06	4.89***
*R* ^*2*^	0.55			0.20		
*F*	67.77***			15.44***		

*Note:* EL = Empowering leadership, EU = Environmental Uncertainty, LMX = Leader‐Member Exchange, UPOB = Unethical Pro‐organizational Behavior. The standardized coefficient is shown in the table.

** *p* < 0.01.

*** *p* < 0.001.

**FIGURE 3:**
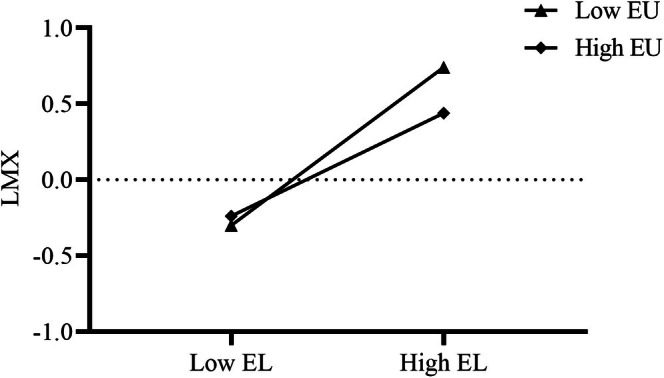
Simple slope analysis. EL = Empowering leadership, EU = Environmental Uncertainty, LMX = Leader‐Member Exchange.

To further explore how EU affects the relationship between EL and LMX, a simple slope analysis of the moderating effect was performed (see Figure 3). It was found that with an increase in EU, the positive influence of EL on LMX weakened (*M‐1SD*: β = 0.21, SE = 0.06, 95%CI = [0.11, 0.33]; *M + 1SD*: β = 0.15, SE = 0.04, 95%CI = [0.07, 0.25]), verifying H3.

## 4 Discussion

Based on the social exchange perspective, this research discussed the effect of EL on UPOB, and explored the roles of LMX and EU in this effect. Overall, our results generally supported our hypotheses. We found that there was a positive relationship between EL and UPOB, and LMX played a mediating role in this relationship. EL tends to give employees more autonomy and provide opportunities for them to participate in decision‐making (Cheong et al. 2019). These behaviors are seen by employees as a sign of trust and respect from the leader, which leads to gratitude towards the leader and a desire to build a closer, long‐term friendly relationship (LMX). Employees are also motivated to complete their role‐based tasks and even go beyond their roles to repay the leader (Umphress and Bingham 2011). However, as pointed out by Cheong et al. (2016), EL not only has positive effects but also poses a burden and pressure on employees. On the one hand, in order to repay the leader, employees are willing to take risks and engage in UPOB (Umphress and Bingham 2011). On the other hand, the existence of the moral licensing effect weakens employees' moral concepts and encourages them to engage in some deviant behaviors such as UPOB (Mishra et al. 2021).

Additionally, the result showed that EU played a moderating role between EL and LMX. An uncertain and ambiguous business environment obviously brings challenges to the effectiveness of leadership. In this context, it is necessary to explore the role of EU. Individuals are inherently averse to uncertainty because it represents a lack of control, which can cause anxiety and stress (Nau 2006). As for employees, when they perceive a high level of EU, they may feel insecure and want to find support and instruction from their leaders (Glambek et al. 2023; Twyford and Le Fevre 2019). In this case, EL empowering employees can seem counterproductive, as employees are not clear on how they should act in this uncertain environment with ambiguous information. Consequently, from the perspective of social exchange, if employees cannot receive support from EL, their emotional attachment and feedback to EL will decrease, thus weakening EL's impact on LMX.

### 4.1 Theoretical Contribution

First, from the perspective of social exchange, this study delves into how EL can influence employee UPOB through LMX. The social exchange theory posits that the relationship between leaders and employees is based on mutual trust and support, and that employees will repay the leader's trust by working hard or engaging in extra‐role behaviors. However, this study further reveals that, employees may take not only positive actions but also certain morally risky behaviors (such as UPOB) to repay the leader. This perspective enriches the existing research on social exchange theory, pointing out the complexity and double‐edged sword effects of social exchange processes in empowering leadership contexts, where granting employees more autonomy may also bring certain moral dilemmas.

Second, this study is the first to explore the impact of EU on the effectiveness of EL, filling a critical gap in current research. Traditionally, most EL studies have focused on its positive effects on employee job satisfaction and performance, but have neglected the constraining role of different environmental factors. This study extends our understanding of the challenges that EL may face in uncertain contexts by revealing the moderating role of EU. This finding not only enriches the theoretical framework of EL but also calls for future research to pay attention to the influence of situational factors on leadership style effectiveness.

### 4.2 Management Implication

Based on the research findings, here are some management recommendations. First, the organization needs to take a series of measures for employees' UPOB, including: establishing an ethically oriented performance evaluation mechanism and incorporating ethical behaviors in employees' work into core performance assessment indicators, in order to reduce their undesirable behaviors in pursuit of performance; establishing a clear and secure reporting mechanism, and setting up a standardized UPOB reporting mechanism (dedicated online platform, independent supervision department, clear feedback time limit, etc.); promoting a leadership style that combines ethical leadership with empowerment, on the one hand, strengthens employees' ethical behavior through demonstration effects, and on the other hand, grants employees a certain degree of autonomy.

Second, leaders should adopt an adaptive leadership strategy: flexibly adjusting their leadership style based on the uncertain environment. For example, in highly uncertain environments, leaders should adjust their leadership style in a timely manner, providing more guidance and support, rather than simply delegating authority to employees. By enhancing the leadership's decision support and communication in ambiguous situations, employees can feel more security and clarity, thereby maintaining a good LMX.

Third, organizations should enhance the level of information sharing to reduce employees' sense of uncertainty. Organizations can achieve this by increasing the transparency of information and ensuring that employees have timely access to the challenges the organization is facing and the strategies being implemented to address them. For example, regular all‐staff meetings, internal reports, or leadership communication can reduce information asymmetry between the organization and employees, enhance employees' understanding of the current situation, and thus maintain confidence and stability in an uncertain environment.

### 4.3 Limitation and Future Prospect

Despite the significant implications of our findings in this era of ever‐changing business, our study has certain limitations that warrant attention and suggest avenues for future research. First, the sample has limitations. In the future, the exploration of the sample in different regions, countries, and different types of enterprises can be expanded to increase the reliability of the results. Second, this study adopted the questionnaire method, which is difficult to reveal the true causal relationship. In the future, the experimental method can be used to further explore the relationship between variables, especially the possible influence relationship between EU and EL. This point needs to be clarified in the future. Third, while this study built on the findings of Cheong et al. (2016) to further explore the burden process of EL, some researchers argue that this impact may not necessarily be linear. For example, Lee et al. (2017) have employed a nonlinear and inverted U‐shaped model to address the too‐much‐of‐a‐good‐thing effect of EL. Therefore, future research should explore the nonlinear relationship between EL and employees' psychology and behavior.

## Ethics Statement

All procedures involving human participants in this study were performed in accordance with ethical standards and with the 1964 Helsinki Declaration and its later amendments or comparable ethical standards. This study was approved by Zhejiang University Ethics Review Board (No. 20220210012).

## Conflicts of Interest

The authors declare no conflicts of interest.

## Data Availability

The data that support the findings of this study are available from the corresponding author upon reasonable request.

## Appendix A

**TABLE A1:** Participant demographics.

Variables	Total	
	No	%
Gender		
Male	282	65.4
Female	149	34.6
Age group		
Between 18 and 25 years	42	9.7
Between 26 and 30 years	65	15.1
Between 31 and 40 years	189	43.9
Between 41 and 50 years	76	17.6
51 years and more	59	13.7
Education		
Secondary education and below	15	3.5
Senior high school education	255	59.2
Bachelor education and above	161	37.4
Position		
No title	84	19.5
Primary title	121	28.1
Intermediate title	146	33.9
Senior title	80	18.6
Working duration with the superior		
Less than 1 years	126	29.2
Between 1 and 3 years	153	35.5
Between 3 and 5 years	137	31.7
More than 5 years	15	3.6
